# Cellular Viability of Partial Heart Transplant Grafts in Cold Storage

**DOI:** 10.3389/fsurg.2021.676739

**Published:** 2021-07-13

**Authors:** Jennie H. Kwon, Morgan Ashley Hill, Brielle Gerry, Jordan Morningstar, Minoo N. Kavarana, Satish N. Nadig, Taufiek Konrad Rajab

**Affiliations:** ^1^Department of Surgery, Medical University of South Carolina, Charleston, SC, United States; ^2^Department of Anatomy and Cell Biology, Medical University of South Carolina, Charleston, SC, United States

**Keywords:** partial heart transplantation, heart valve transplantation, heart valve replacement, pediatric cardiac surgery, viability, cold storage

## Abstract

Congenital heart defects are the most common types of birth defects in humans. Children with congenital heart defects frequently require heart valve replacement with an implant. Unfortunately, conventional heart valve implants do not grow. Therefore, these children are committed to serial re-operations for successively larger implant exchanges. Partial heart transplantation is a new and innovative approach to deliver growing heart valve implants. However, the transplant biology of partial heart transplant grafts remains unexplored. This is a critical barrier for clinical translation. Therefore, we investigated the cellular viability of partial heart transplants in cold storage. Histology and immunohistochemistry revealed no morphological differences in heart valves after 6, 24, or 48 h of cold storage. Moreover, immunohistochemistry showed that the marker for apoptosis activated caspase 3 and the marker for cell division Ki67 remained unchanged after 48 h of cold storage. Finally, quantification of fluorescing resorufin showed no statistically significant decrease in cellular metabolic activity in heart valves after 48 h of cold storage. We conclude that partial heart transplants remain viable after 48 h of cold storage. These findings represent the first step toward translating partial heart transplantation from the bench to the bedside because they have direct clinical implications for the procurement logistics of this new type of transplant.

## Introduction

Congenital heart defects are the most common type of birth defects in humans. In North America, 7 in 1,000 live born children are affected (1). Worldwide, this causes over 180,000 infant deaths per year (2). Treatment of congenital heart defects frequently involves heart valve replacement with an implant (3). However, conventional heart valve implants do not grow with recipient children. These children are committed to serial re-operations for successively larger heart valve implant exchanges (4). Therefore, an intensive research effort is focused on delivering growing heart valve replacements for children with congenital heart defects.

**Figure 1:**
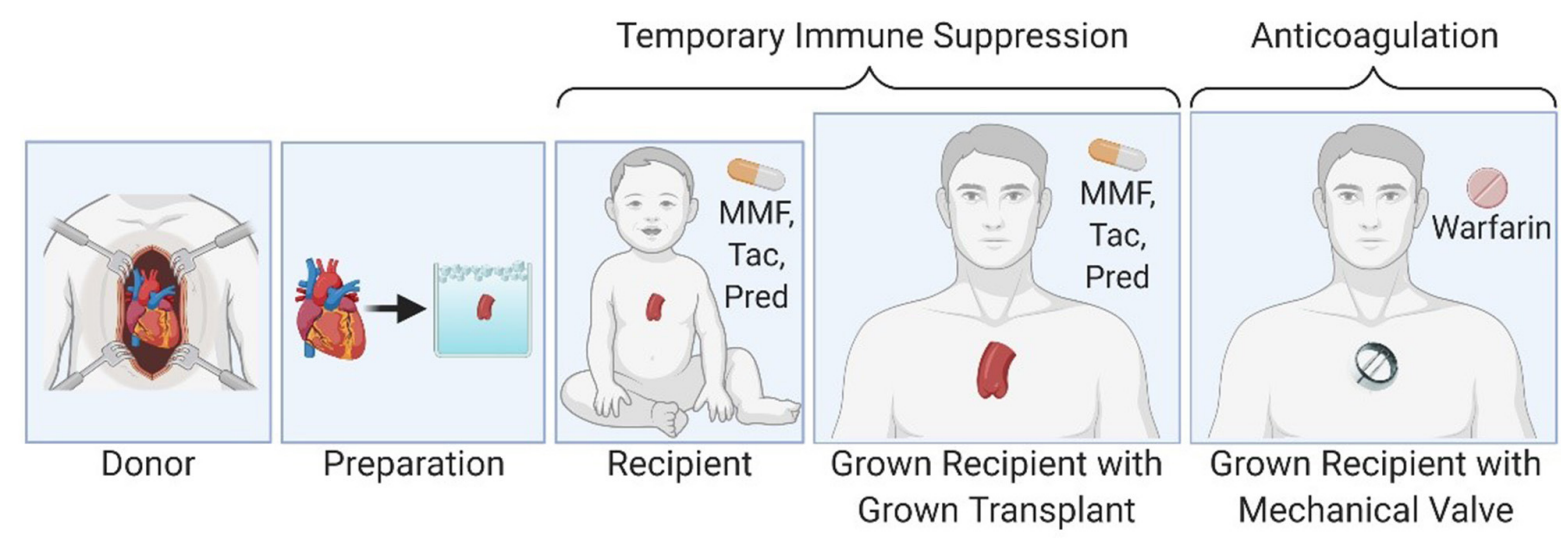
Partial heart transplantation involves transplantation of a heart valve and temporary immune suppression until the transplanted valve can be exchanged for an adult-sized prosthetic valve in the grown child.

Conventional approaches to deliver growing heart valve replacement are based on tissue engineering or mechanical engineering but these approaches have failed in clinical translation (5–9). This is a critical barrier to progress in the field. To overcome this barrier, we propose partial heart transplantation as a new approach to deliver a growing heart valve replacement with the ability to self-repair and avoid thrombogenesis (Figure 1). This involves transplantation of a heart valve only (10). The transplanted heart valve will grow with the recipient child, just like a conventional heart transplant or a Ross pulmonary autograft (11–13). This new type of transplant is readily translatable to the clinic because it resembles conventional heart transplantation from a transplant immunology perspective. However, there are important distinctions between conventional heart transplants and partial heart transplants. For example, conventional heart transplants must perform metabolically demanding work immediately after implantation, whereas partial heart transplants primarily serve a structural function. Therefore, we hypothesized that partial heart transplant grafts tolerate a substantially longer cold storage time. For example, a 48-h cold storage time would allow for convenient delivery of the donor graft using commercial couriers anywhere in the continental United States. Here, we examine the cellular viability of partial heart transplant donor grafts in cold storage for 48 h.

## Methods

### Heart Valve Tissue

**Figure 2:**
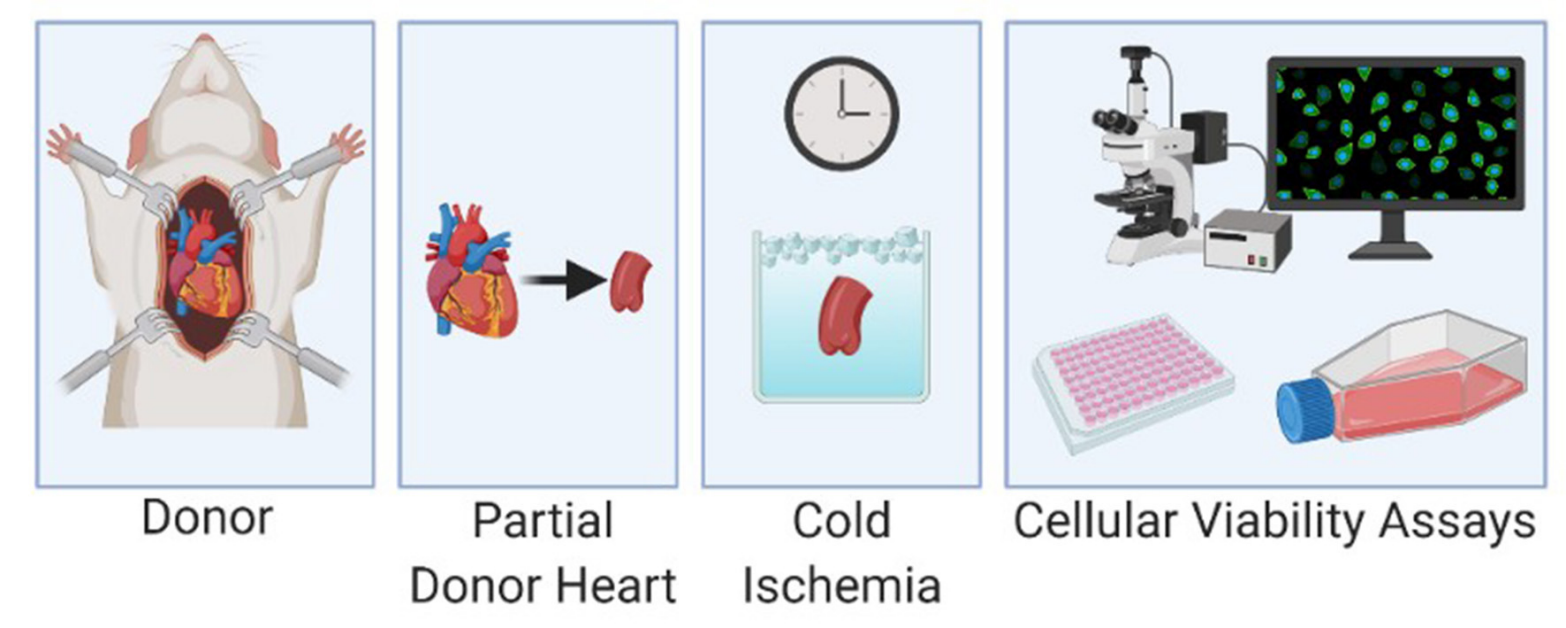
Experimental protocol with harvest of the donor aorta, cold storage of the specimen and cellular viability analysis.

Heart valve tissue from rats was obtained from hearts procured by the usual protocol for heart transplantation (IACUC protocol 2020-01093). Briefly Sprague Dawley rats (Charles River Laboratories, Wilmingon, MA) were anesthetized with isoflurane. A sternectomy was performed to access the heart. The animals were exsanguinated, the aorta was cross-clamped and the heart flushed with ice-cold University of Wisconsin solution (Global Transplant Solutions, SC). The hearts were then excised by dividing the inferior vena cava, left atrium, superior vena cava, pulmonary artery and aorta. The donor aortic valve was harvested from the donor hearts and stored in ice-cold University of Wisconsin solution until cellular analysis (Figure 2).

### Cold Storage

Experience with conventional heart transplants shows that a cold-preservation time of 6 h has no effect on the growth or function of the transplanted valves (14, 15). Therefore, cold storage for 6 h served as a negative control. After a predetermined interval in cold storage, the heart valves were analyzed as described below.

### Cellular Viability Analysis

Tissue structure was analyzed by histology with hematoxylin and eosin stain, Masson trichrome stain, and immunohistochemistry for aSMA and CD31.

Cellular apoptosis was analyzed by immunohistochemistry for activated caspase 3.

Cellular division was analyzed by immunohistochemistry for Ki67.

Cellular metabolic activity within aortic valve grafts was measured by quantification of fluorescing resorufin, which is produced by cellular reduction of resazurin. Whole aortic valve grafts were stored in University of Wisconsin buffer for 6, 24, and 48 h then rinsed in PBS and incubated in DMEM with 1% antibiotic/antimycotic solution and 10% AlamarBlue reagent (Invitrogen, CA) at 37 degrees Celsius for 1 h. Resorufin fluorescence was measured at an excitation wavelength of 545 nm with exposure time 1/15 s (Keyence, Japan), and measured fluorescence intensity was normalized to tissue mass. Three replicate aortic samples were measured for each cold ischemia time period.

## Results

### Tissue Structure

**Figure 3:**
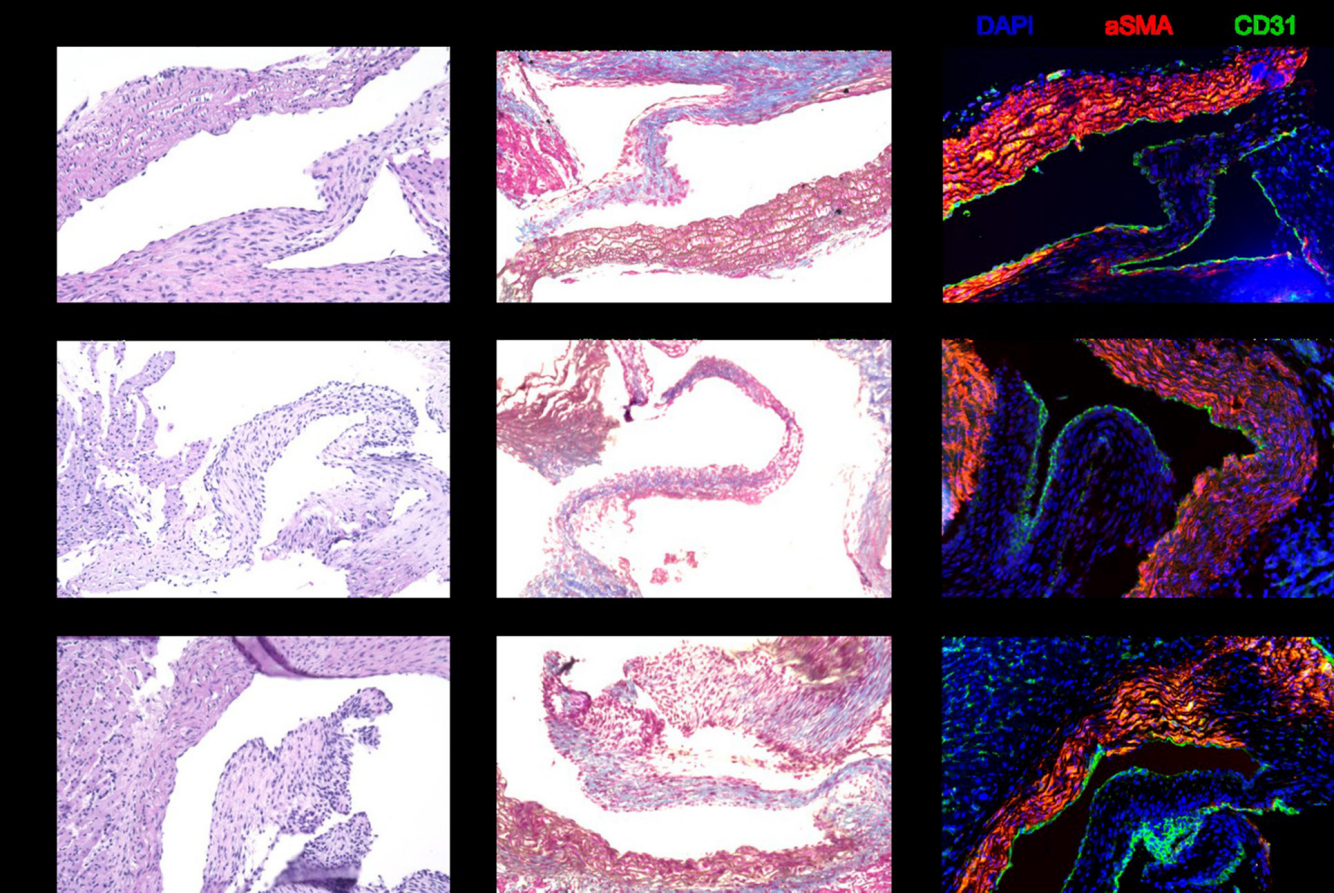
Representative sections stained with hematoxylin and eosin, Masson trichrome, and immunohistochemistry for DAPI, aSMA, and CD31. There are no structural differences in the donor heart valves between 6, 24, and 48 h of cold storage.

Histology with hematoxylin and eosin stain and Masson trichrome stain revealed no morphological differences in heart valves after 6, 24, or 48 h of cold storage. The valve leaflets were structurally intact, and there was no evidence of edematous swelling (Figure 3). Structural integrity of the heart valves was confirmed by immunohistochemistry for aSMA and CD31 (Figure 3).

### Apoptosis and Cell Division

**Figure 4:**
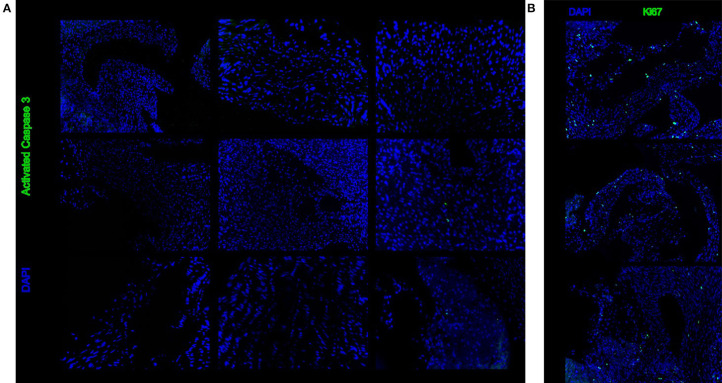
Representative sections stained with immunohistochemistry for activated caspase 3 **(A)** or Ki67 **(B)**. There is no difference between 6, 24, and 48 h of cold storage.

Immunohistochemistry for activated caspase 3 showed that there is no increased level of apoptosis in heart valves after 48 h of cold storage (Figure 4A). Similarly, immunohistochemistry for Ki67 showed that markers of cell division were unchanged after 48 h of cold storage (Figure 4B).

### Cellular Metabolic Activity

**Figure 5:**
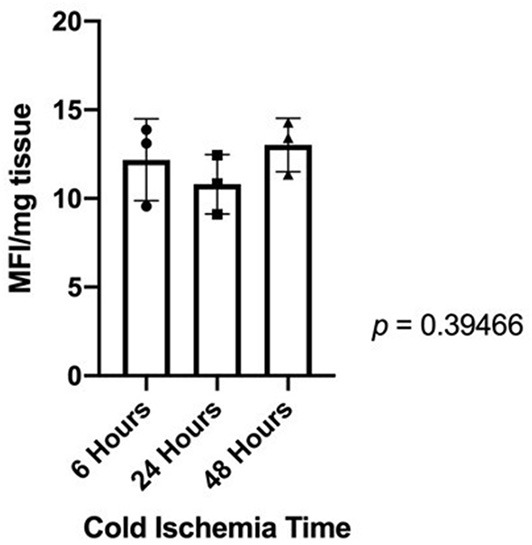
Fluorescing resorufin quantification showed that there is no statistically significant difference in cellular metabolic activity between donor heart valves in cold storage for 6, 24, or 48 h.

Quantification of fluorescing resorufin showed no statistically significant decrease in cellular metabolic activity in heart valves after 48 h of cold storage (Figure 5).

## Discussion

Partial heart transplantation is a new concept to deliver growing heart valve implants for children. It readily translatable both from a surgical perspective and from a transplant immunology perspective. However, the detailed transplant biology for this new type of transplant remains unexplored.

Here we show that the cellular viability of heart valve transplants remains stable within 48 h of cold storage. This finding has important clinical implications for the logistics of partial heart transplantation. Importantly, shipping of the donor tissue with a commercial courier likely is an option in the continental United States. This contrasts which conventional heart transplants, which require dedicated procurement teams to limit the cold storage time to 6 h. Moreover, the procurement radius for partial heart transplants is significantly expanded compared to conventional heart transplants.

The current study is limited to an examination of cellular viability of the partial heart transplant donor grafts. In addition to decreased cellular viability during cold storage, ischemia reperfusion injury contributes to the success of transplantation. Therefore, future studies are necessary to investigate the effects of ischemia reperfusion injury on partial heart transplants and potentially identify specific ways to mitigate it.

In conclusion, our findings that structural integrity and cellular viability persist in partial heart transplant grafts support the feasibility of partial heart transplantation to deliver growing heart valve replacements for children. Moreover, the data presented here will help with the design of the procurement protocol for a pilot clinical trial for heart valve transplantation.

## Author Contributions

All authors listed have made a substantial, direct and intellectual contribution to the work, and approved it for publication.

## Conflict of Interest

TKR discloses a preliminary patent relevant to partial heart transplantation. The remaining authors declare that the research was conducted in the absence of any commercial or financial relationships that could be construed as a potential conflict of interest.
